# The 10-Year Study of the Impact of Particulate Matters on Mortality in Two Transit Cities in North-Eastern Poland (PL-PARTICLES)

**DOI:** 10.3390/jcm9113445

**Published:** 2020-10-27

**Authors:** Łukasz Kuźma, Emil Julian Dąbrowski, Anna Kurasz, Hanna Bachórzewska-Gajewska, Sławomir Dobrzycki

**Affiliations:** 1 Department of Invasive Cardiology, Medical University of Bialystok, 24A Sklodowskiej-Curie St, 15-276 Bialystok, Poland; e.j.dabrowski@gmail.com (E.J.D.); annaxkurasz@gmail.com (A.K.); hgajewska@op.pl (H.B.-G.); kki@umb.edu.pl (S.D.); 2 Department of Clinical Medicine, Medical University of Bialystok, 24A Sklodowskiej-Curie St, 15-276 Bialystok, Poland

**Keywords:** air pollution, mortality, particulate matter

## Abstract

The detrimental influence of air pollution on mortality has been established in a series of studies. The majority of them were conducted in large, highly polluted cities—there is a lack of studies from small, relatively clean regions. The aim was to analyze the short-term impact of particulate matters (PMs) on mortality in north-eastern Poland. Time-stratified case-crossover design was performed for mortality in years 2008–2017. Daily concentrations of PM_2.5_ (28.4 µg/m^3^, interquartile range (IQR) = 25.2) vs. (12.6 µg/m^3^, IQR = 9.0) and PM_10_ (29.0 µg/m^3^, IQR = 18.0) vs. (21.7 µg/m^3^, IQR = 14.5) were higher in Łomża than Suwałki (*p* < 0.001). Impact of PM_2.5_ on mortality was recorded in Łomża (odds ratio (OR) for IQR increase 1.061, 1.017–1.105, *p* = 0.06, lag 0) and Suwałki (OR for IQR increase 1.044, 1.001–1.089, *p* = 0.004, lag 0). PM_10_ had an impact on mortality in Łomża (OR for IQR increase 1.028, 1.000–1.058, *p* = 0.049, lag 1). Cardiovascular mortality was affected by increase of PM_2.5_ in Łomża (1.086, 1.020–1.156, *p* = 0.01) and Suwałki (1.085, 1.005–1.171, *p* = 0.04). PM_2.5_ had an influence on respiratory mortality in Łomża (1.163, 1.021–1.380, *p* = 0.03, lag 1). In the whole studied region, despite differences in the air quality, the influence of PMs on mortality was observed.

## 1. Introduction

The detrimental influence of air pollution on health and mortality have been established in a series of studies by researchers all over the world [1,2,3]. Long-term as well as short-term exposure contributes to the development or progression of coronary artery disease, cardiac arrhythmias, heart failure, chronic obstructive pulmonary disease, and many others, with a significant impact on both life quality and life expectancy [4,5,6,7]. According to data from the Global Burden of Diseases 2017 Study, outdoor and indoor air pollution contributes to almost 5 million deaths per year [8].

Air pollution is a complex mixture of particles and gases that can be man-made (anthropogenic) as well as come from natural sources. Common air pollutants are carbon monoxide (CO), particulate matter (PM), lead (Pb), ozone (O_3_), nitrogen dioxide (NO_2_), and sulfur dioxide (SO_2_). PM is typically defined by size—particles with a diameter of 10 μm or less are called PM_10_ and with a diameter of 2.5 μm or less PM_2.5_. We can also distinguish ultrafine particles with a diameter below 0.1 μm.

Over the years, changes in systemic regulations have been introduced in developed countries that have led to improvements in air quality, but even when the level of pollutants is below current targets, their impact on mortality is still visible. Air pollution-associated health effects are particularly pronounced in developing countries [9].

A vast amount of research focuses on highly polluted agglomerations which are an adequate place to conduct such research due to the widespread presence of an air quality monitoring system. The common belief in clean air in relatively small towns, often near rural regions, may be incorrect and have its source in the scarce number of permanent large-scale measuring stations in these areas, which is reflected in our study. Waste incineration as well as the quality of fuels used in household furnaces is not without significance. Low environmental awareness and socioeconomic conditions contribute to the deepening of the problem.

Taking this into consideration we decided to analyze clinical data with both air quality and meteorological data from two small cities over a period of 10 years.

## 2. Patients and Methods

### 2.1. Study Design

Data on mortality were collected from the National Statistical Office in Poland. The records include the information on all the deaths with the age and sex recorded in Łomża (id commune 206301) and Suwałki (id commune 200702) in the years 2008–2017. According to codes in the International Classification of Diseases-10th Revision, we extracted the data for cardiovascular-related mortality (ICD-10 from I.00 to I.99) and pulmonary-related mortality (ICD-10 from J.00 to J.99).

**Figure 1:**
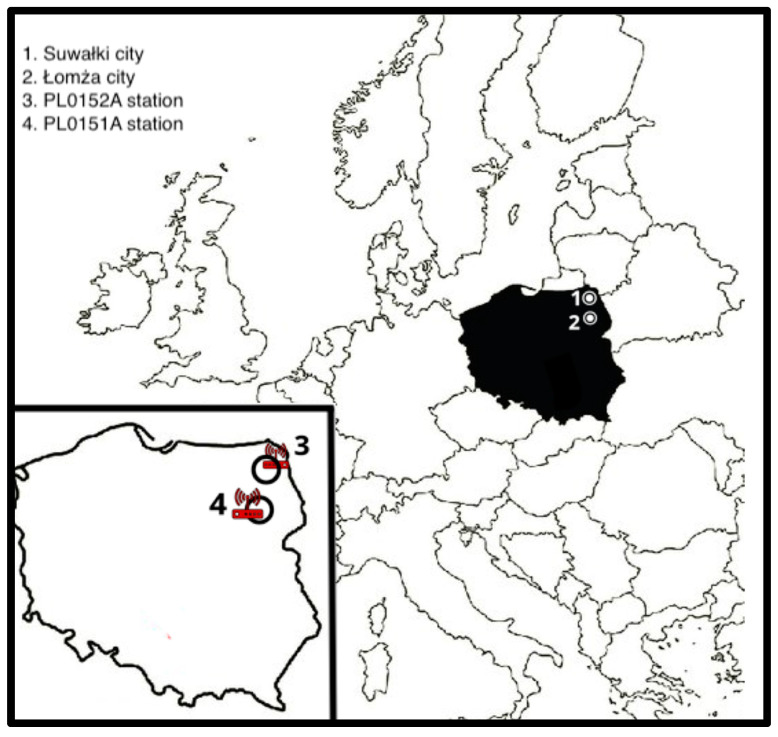
Characteristics of the studied cites.

Standardized death rates (SDR) were calculated based on the standard European population structure [10]. The data of air pollutions and temperature were obtained from Voivodeship Inspectorate for Environmental Protection in Bialystok and the Institute of Meteorology and Water Management. In the analysis, we used the concentration of particulate matter with a diameter of 2.5 µm or less (PM_2.5_), 10 µm or less (PM_10_), and temperature. In Łomża city, all measurements (PM_2.5_, PM_10_) were obtained from a station located in the northern part of the city-PL0151A (GPS: 53°18′ N, 22°05′ E). The temperature measurements were obtained for years 2008–2011 from station ID 253210210 (GPS: 53°39′ N, 21°34′ E), for 2011–2015 (ID 253220280; GPS: 53°24′ N, 22°15′ E) and station 253220330 (GPS 53°21′ N, 22°10′ E) for 2016 and 2017. In Suwałki, the measurements of air pollution were obtained from station PL0152A (GPS: 54°12′ N, 22°93′ E), the temperature from the station ID 354220195 (GPS: 54°13′ N, 22°95′ E) (Figure 1).

The 24 h mean daily values of physical data were used for statistical analysis.

The exceedance of air pollution norms was determined based on the World Health Organization guidelines concerning air quality. The 24-h concentrations recommended by the WHO are 50 µg/m^3^, 25 µg/m^3^ for PM_10_, PM_2.5_, respectively [11]. The study material lacked about 14.1% in Łomża and 24.49% in Suwałki. Days with missing data were excluded from the analysis.

### 2.2. Region′s Characteristics

Both of the analyzed cities are located in Podlaskie Voivodeship-north–eastern part of Poland widely known as the Green Lungs of Poland. The name of the region is derived from the surrounding of national parks, lack of factories, and relatively low industrialization. However, despite their unique location, the characteristics of both cities contribute to an increased level of air pollution. Suwałki and Łomża take an important part in the transit traffic from Northern and Eastern Europe to Central Europe and during the study, neither of the cities had ring roads. Both cities have a similar population, percentages of people over 65 years old with 15.1% in Suwałki and 16.7% in Łomża, and femininity ratio of 109 in Suwałki and 110 in Łomża.

### 2.3. Statistical Analysis

The distribution of variables was evaluated using the Kolmogorov–Smirnov test. Variables were expressed as mean values with standard deviation. A two-tailed t-test was used for comparative analysis. Spearman correlation test was applied for evaluating the relationships between the levels of PMs and temperature. Results are reported as r score and plots.

To assess the effect of particulate matter on mortality we used a time-stratified case-crossover study design [12,13]. The day of death was defined as the case period, while control periods included all days that were from the same day of the week in the same month as the case period. Because of high collinearity, to minimize that effect, each air pollutant was modeled individually. Separate models were created for temperature.

The association between PMs and the occurrence of deaths was estimated by odds ratios (ORs) with 95% confidence intervals (CIs) using conditional logistic regression (CLR). Meteorological data including temperature during the same lag period were used as covariates in the CLR model. We used a natural cubic spline with 5 degrees of freedom for the temperature–mortality function. We made separated models for total mortality, cardiovascular mortality and pulmonary mortality.

Results are reported as OR associated with an increase in interquartile range (IQR) at days with lag from 0 to 2.

The differences in the odds ratio between cities were calculated according to the Altman method [14]. The results were presented as ratios of OR (ROR) and CI from the 5th to the 95th percentile.

All analyses were performed using MS Excel (Microsoft, 2020, version 16.40, Redmond, WA, USA) and XL Stat (Addinsoft, 2020, version 2020.03.01, New York, NY, USA). The study protocol conformed to the ethical guidelines of the 1975 Declaration of Helsinki, STROBE guidelines [15], and was approved by the local bioethics committee of the Medical University of Bialystok (Approval No. R-1-002/18/2019) and registered in the database of clinical studies www.clinicaltrials.gov (Identifier: NCT04541498).

The two-tailed *p*-value < 0.05 was considered statistically significant.

## 3. Results

From 2008 to 2017 we recorded 7486 deaths in Suwałki and 8082 in Łomża. The male sex was dominant in both of the cities—54.1% in Suwałki, 54.5% in Łomża. The mean age of deaths in Suwałki was 71.7 years (SD (standard deviation) = 16.6), in Łomża—72.7 years (SD = 15.7), (*p* < 0.001). The crude death rate (CDR) (1288.1 per 100,000 population/year) as well as standardized death rate (SDR) (1944.6 per 100,000 population/year) was the highest in Łomża (Table 1).

In Suwałki, more prevalent causes of death were malignant neoplasm of bronchus and lung (8.0% (N = 597) vs. 6.4% (N = 518), *p* < 0.001), malignant neoplasm of breast (2.1% (N = 157) vs. 1.6% (N = 127); *p* = 0.014), hypertensive heart disease (2.2% (N = 168) vs. 1.8% (N = 144); *p* = 0.040), and unspecified heart disease (5.0% (N = 372) vs. 3.5% (N = 285); *p* < 0.001). Heart failure (3.4% (N = 276) vs. 1.9% (N = 141); *p* < 0.001), cerebral infarction (9.2% (N = 744) vs. 5.8% (N = 432), *p* < 0.001), myocardial infarction (3.9% (N = 315) vs. 3.1% (N = 232); *p* = 0.007), and chronic obstructive pulmonary disease (3.2% (N = 258) vs. 2.6% (N = 193); *p* = 0.022) were more frequent causes of deaths in Łomża (Table 2).

**Figure 2:**
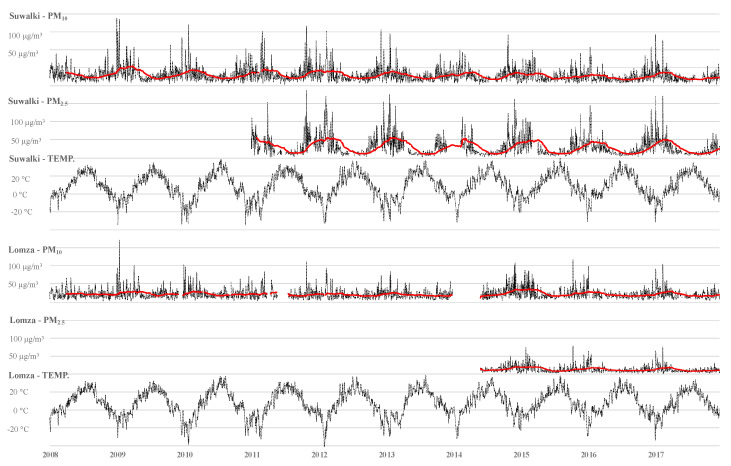
Panel chart. Changes in the concentrations of air pollutants and temperature in studied cities for analyzed period (the red line represents changes in the quartile of the year).

Over the observation time, daily mean concentrations of PM_2.5_ and PM_10_ were analyzed as well as the mean levels of temperature. In each of the analyzed years, concentrations of PM_2.5_ and PM_10_ were higher in Łomża. The daily concentrations of PM_2.5_ (28.4 µg/m^3^, SD = 24.3, IQR = 25.2) vs. (12.6 µg/m^3^, SD = 9.2, IQR = 9.0) and PM_10_ (29.0 µg/m^3^, SD = 19.4, IQR = 18.0) vs. (21.7 µg/m^3^, SD = 14.0, IQR = 14.5 were higher in Łomża than Suwałki (*p* < 0.001) (Table 3). The limit of the daily mean given by the WHO guidelines for PM_2.5_ in Łomża and Suwałki was exceeded on 40.7% and 8.4% of days, respectively. The daily WHO upper limit for PM_10_ was exceeded on 9.8% of days in Łomża and 4.2% in Suwałki. The daily mean temperature in Łomża was 7.9 °C (SD = 8.8) and in Suwałki it was 7.4 °C (8.8) (Table 3 and Figure 2).

A strong positive correlation was found between concentration of particulate matters-PM_2.5_ vs. PM_10_ (r = 0.668, *p* < 0.001) in Suwałki. A strong negative correlation was observed in Łomża between PM_2.5_ vs. temperature (r = −0.608, *p* < 0.001) alongside moderate positive PM_2.5_ vs. PM_10_ correlation (r = 0.518, *p* < 0.001) (Table 4).

The impact of PM_2.5_ on total mortality was recorded in Suwałki (OR for IQR increase 1.044, 95% CI 1.001–1.0.89, *p* = 0.04) and Łomża (OR for IQR increase 1.061, 95%CI 1.017–1.105, *p* = 0.006). The effect of PM_10_ was observed in Łomża on lag 1 (OR for IQR increase 1.028, 95% CI 1.000–1.058, *p* = 0.049) and lag 2 (OR for IQR increase 1.030, 95% CI 1.001–1.060, *p* = 0.04). In case of Suwałki the effect of PM_10_ was observed at lag 2 OR = 1.034 (95% CI 1.005–1.064, *p* = 0.02).

The effect of PMs on cardiovascular mortality was noted in Suwałki on lag 0 OR = 1.085 (95% CI 1.005–1.171, *p* = 0.037) for PM_2.5_ and OR = 1.056 (95% CI 1.006–1.07, *p* = 0.03) for PM_10_. Moreover, in Łomża, the effect of PM_2.5_ was also noted (OR 1.086, 95% CI 1.020–1.156, *p* = 0.01, lag 0).

In the analysis of pulmonary mortality, only the effect of PM_10_ on lag 1 was noted in Łomża (OR = 1.163 95% Cl 1.021–1.380). No differences in the impact of air pollution on mortality were observed between both cities (Table 5).

## 4. Discussion

The majority of studies that take up the matter of air pollution are conducted in big cities, often in Chinese megalopolises and densely populated Indian agglomerations. This leads to the marginalization of the problem in smaller cities. However, if we compare SDRs in Suwałki and Łomża with the mean SDR for Poland (1638 and 1945 vs. 1218 per 100,000 inhabitants), we come to the realization that there are no reasons for this negligence [16].

Major findings of the study are the following: in both of the cities, despite differences in air quality, the influence of PMs on total and cardiovascular mortality was observed, while PM_2.5_ was associated with pulmonary mortality in Łomża. Different lag patterns were observed for each of the cities.

The origin of air pollutants has an impact on the chemicals that are bound on the surface of particulate matter [17,18]. When comparing the health effects of various PM_2.5_ sources, diesel exhaust particles (DEPs) represent the highest mutagenic activity, generate the most intracellular reactive oxygen species (ROS), and cause altered vascular transcription [19,20,21]. Generation of ROS is crucial and influences both long- and short-term effects of air pollutants.

The burden of diesel exhaust particles was exposed by Yorifuji et al. in their quasi-experimental study. In 2003 in Tokyo, diesel emission control ordinance was introduced, forcing diesel vehicles to meet the norms for PM. It resulted in a spectacular 44% decrease in PM_2.5_. Compared to Osaka, which introduced similar regulations in 2009, there was a decrease in cardiovascular mortality of 11%, ischemic heart disease mortality of 10%, and cerebrovascular disease mortality of 6.2% [22].

The topic of poor air quality in smaller cities is a multi-level issue. Firstly, in both analyzed cities, due to the lack of ring roads, major national trunk roads run through the city centers. Traffic lights, crossroads, pedestrian crossings, and heavy traffic during peak hours extend the time of large goods vehicles spent on passing the city. Secondly, when comparing highly urbanized cities with more rural ones, the higher prevalence of detached houses in the latter is apparent. It has been proved in numerous studies that air pollutants’ concentrations fluctuate during the year, reaching the highest values during heating seasons. It seems obvious that its peak is linked with coal combustion processes that are more intensified in housing estates. The third issue is the lack of air quality monitoring stations in smaller cities. In Suwałki, PM_2.5_ concentration measurements started only in 2014, and in September 2020 there are still no gaseous pollutants concentration measurements stations. Lastly, the favoring of coal-fired power plants by authorities is an important obstacle on the way to the cleaner air.

Łomża represented over twice higher mean daily concentrations of PM_2.5_ than Suwałki (28.4 vs. 12.6 µg/m^3^) and exceeded mean daily WHO guidelines almost five times more frequently (40.7% vs. 8.4%). When analyzing PM_10_ concentrations, although differences in values were not so highly demonstrated, they remain higher in Łomża (21.7 vs. 29.0 µg/m^3^).

When comparing the contributions of cause-specific mortality in all-cause mortality between two cities, the most apparent disbalance lies in the prevalence of cerebral infarctions. The more polluted city, Łomża demonstrated almost twice higher cerebral infarction mortality rate than Suwałki (9.2% vs. 5.8%). This comes in line with many studies reporting the link between an increase in particulate matter concentrations and the risk of cerebrovascular events [23,24]. Referring to research, PM_2.5_ has a greater impact on mortality than PM_10_ [25]. Shi et al. in their analysis of PM_2.5_-induced premature mortality reported that stroke was the most important cause, accounting for 39% of the total mortality [26]. The mechanisms of influence are not fully understood yet, however few pathways are considered to be possible. They include endothelial dysfunction, systemic inflammation, rheological parameter alternations, and formation of thrombi as a result of atrial fibrillation induction [27,28].

Many studies from the past provided strong evidence on particulate matter influence on cardiovascular mortality [29,30]. It was reported that an increase in 10 µg/m^3^ of PM_2.5_ and PM_10_ concentrations increased daily cardiovascular mortality rate by 0.55% and 0.36%, respectively [31]. Reported associations were stronger especially in cities with lower annual mean particulate matter concentrations. As we expected, our study proved that PMs have an influence on cardiovascular mortality. PM_2.5_ was responsible for CVD mortality in both of the analyzed cities. However, PM_10_ manifested its influence only in less polluted of the analyzed cities (Suwałki). No lag effect was observed.

It is widely acknowledged that PM_2.5_ is more harmful for the respiratory system than PM_10_ [32]. Smaller size permits deeper inhalation into small alveoli, resulting in greater surface of interaction. Induction of inflammation and generation of ROS seems to be crucial in a detrimental influence on the respiratory system [33]. The most recent hypotheses put in the spotlight the influence of PM_2.5_ on airway microbiome profile and alternation of pulmonary function [34]. Interestingly, meta-analysis of PM_2.5_ and daily mortality revealed that a 10 µg/m^3^ increment was responsible for a higher risk of death due to respiratory rather than cardiovascular causes (1.51% vs. 0.84%) [35]. In our study, IQR increase in exposure to PM_2.5_ concentrations had an impact on mortality in Łomża only on the day after exposure. Some studies claim that the early lag effect is more specific for cardiovascular diseases, whereas the prolonged lag effect is linked with respiratory diseases, especially asthma [36]. Similar results were reported in a research conducted in Madrid, showing relationship between short-term exposure and increase in all-cause pulmonary mortality on lags 1 and 2 [37].

The effect of IQR increase on PM_2.5_ concentrations on all-cause mortality was similar in both of the cities, showing a statistically significant association only on the day of exposure. When comparing the results with other European studies, we notice some heterogeneity. It has been proved that PM_2.5_ have a significant influence on mortality in Stockholm and the Netherlands [38,39]. In the latter study, the influence on all-cause mortality was reported on all of the analyzed lags. On the other hand, Atkinson et al. found little evidence for associations of PM_2.5_ and all-cause mortality in London [40].

PM_10_ had an influence on all-cause mortality on lag 1 only in Łomża and in both of the cities on lag 2. Similar results were reported in several studies. The study by Choi et al. conducted in Seoul proved that PM_10_ was associated with all-cause and cardiovascular mortalities, however not with respiratory mortalities [41]. The aforementioned Dutch study observed an association between PM_10_, all cause, and respiratory mortality [39]. Recent Sicilian research proved an association between PM_10_, all-cause, and cause-specific mortality [42].

A different pattern of lag effects between cities has been reported in other publications [43]. It could be due to different sources of pollution, resulting in different chemicals bound on the surface of particulate matter [44]. Another hypothesis might include differences in gaseous pollutant concentrations, as the amplifying effect of the harmful influence of gaseous pollutants on particulate matter is well-known [45].

In this study, we used data only from patients registered as residents of Łomża and Suwałki. Having only one monitoring station in each of the cities, it made spatiotemporal variations impossible to analyze, leading to unavoidable exposure misclassification. Both of the monitoring stations lie within the boundaries of the cities. Exposure misclassification bias might have been less significant in Łomża, as the city’s area is over twice as small as Suwałki’s. Yu et al. in their work analyzed the impact of spatiotemporal subject mobility on estimates of ambient air pollutant exposure [46]. They compared home location-based exposure (HBE) and call detail record location-based exposure (CDRE). Mean PM_2.5_ exposure was only 0.4 µg/m^3^ higher in CDRE (72.9 vs. 72.5 µg/m^3^). Taking into consideration the results mentioned above, relatively small areas of the analyzed cities, and the fact that nondifferential misclassification of exposure bias is towards the null association, we expect the alternation of results, especially in Suwałki, but not to the extent that would mask the correct conclusions.

## 5. Conclusions

In the whole studied region despite differences in air quality, the influence of PMs on all-cause mortality was observed. This effect was prolonged up to one and two days after the exposure. Cardiovascular mortality in both of the cities was influenced by PM_2.5_ on lag 0, whereas PM_10_ was associated only with higher mortality rate in Suwałki. Pulmonary mortality rate was associated with increase in PM_2.5_ concentrations only in Łomża on lag 1. Implementation of strategies aimed at reducing exposure to traffic-derived air pollution will contribute to the improvement of cardiovascular and respiratory health.

## 6. Limitations

The main limitation of our study was the lack of gaseous pollutants data. This information could have let us draw better conclusions concerning lag effects and cause-specific mortality. Having only one monitoring station in each of the cities certainly lead to some exposure misclassification. Lastly, case-specific mortality might be underestimated due to garbage codes.

## Figures and Tables

**Table 1:** Mortality data in the studied cities.

	Suwałki	Łomża	*p*
Total deaths, N	7486	8082	
Male, % (N)	54.1 (4055)	54.5 (4408)	0.640
Mean age (SD)	71.7 (16.6)	72.7 (15.7)	<0.001
CDR (100,000 population/year)	1079.5	1288.1	<0.001
SDR (100,000 population/year)	1638.1	1944.6	<0.001

SD, standard deviation; CDR, crude death rate; SDR, standardized death rate.

**Table 2:** Cause-specific mortality in studied cities.

	Suwałki	Łomża	*p*
All, % (N)	100 (7486)	100 (8082)	N/A
Cardiovascular deaths, % (N)	36.4 (2724)	41.2 (3328)	<0.001
Pulmonary deaths, % (N)	7.3 (549)	6.5 (528)	<0.001
Chronic ischemic heart disease, % (N)	8.5 (633)	9.1 (733)	0.176
Cerebral infarction, % (N)	5.8 (432)	9.2 (744)	<0.001
Heart disease-unspecified, % (N)	5.0 (372)	3.5 (285)	<0.001
Myocardial infarction, % (N)	3.1 (232)	3.9 (315)	0.007
Intracerebral hemorrhage, % (N)	2.3 (174)	2.7 (219)	0.126
Hypertensive heart disease, % (N)	2.2 (168)	1.8 (144)	0.040
Heart failure, % (N)	1.9 (141)	3.4 (276)	<0.001
Malignant neoplasm of bronchus and lung, % (N)	8.0 (597)	6.4 (518)	<0.001
Instantaneous death, % (N)	2.6 (196)	2.8 (225)	0.524
Chronic obstructive pulmonary disease, % (N)	2.6 (193)	3.2 (258)	0.022
Pneumonia, % (N)	2.5 (188)	2.3 (183)	0.313
Diabetes mellitus, % (N)	2.1 (160)	2.6 (206)	0.090
Malignant neoplasm of breast, % (N)	2.1 (157)	1.6 (127)	0.014
Malignant neoplasm of colon, % (N)	2.0 (152)	1.9 (152)	0.121
Malignant neoplasm of prostate, % (N)	2.0 (148)	1.7 (137)	0.190
Senility, % (N)	1.9 (139)	2.2 (176)	0.155
Suicide, % (N)	1.8 (132)	0.8 (62)	<0.001
Atherosclerosis, % (N)	1.7 (125)	1.8 (146)	0.515
Malignant neoplasm of gastric, % (N)	1.6 (116)	1.4 (115)	0.514
Other, % (N)	40.5 (3031)	37.9 (3061)	<0.001

N/A; not applicable.

**Table 3:** Statistics for daily concentrations of particulate matters and temperature.

Variables	PM_2.5_ µg/m^3^			PM_10_ µg/m^3^			Temp. °C		
	Suwałki	Łomża	*p*	Suwałki	Łomża	*p*	Suwałki	Łomża	*p*
Days with observation; N, (%)	1309 (35.8)	2230 (61.1)	<0.001	3313 (90.7)	3533 (96.7)	<0.001	3653 (100)	3653 (100)	N/A
2008; mean/day (SD)	N/D	N/D	N/A	21.5 (11.6)	31.2 (19.1)	<0.001	8.0 (7.2)	8.5 (7.3)	0.346
2009; mean/day (SD)	N/D	N/D	N/A	23.7 (18.2)	34.1 (25.1)	<0.001	6.9 (8.6)	7.2 (8.6)	0.559
2010; mean/day (SD)	N/D	N/D	N/A	22.1 (13.3)	29.9 (19.8)	<0.001	6.3 (10.7)	6.5 (10.5)	0.754
2011; mean/day (SD)	N/D	33.02 (25.6)	N/A	21.4 (14.2)	34.0 (23.8)	<0.001	7.4 (9.0)	8.1 (8.9)	0.346
2012; mean/day (SD)	N/D	33.2 (29.4)	N/A	20.2 (12.8)	29.9 (20.1)	<0.001	6.6 (9.8)	7.3 (9.9)	0.350
2013; mean/day (SD)	N/D	27.9 (24.7)	N/A	19.1 (11.2)	27.1 (15.7)	<0.001	7.2 (9.1)	7.7 (9.0)	0.535
2014; mean/day (SD)	15.1 (8.7)	28.0 (24.5)	<0.001	25.9 (16.8)	29.4 (18.0)	0.007	7.8 (8.8)	8.2 (8.7)	0.551
2015; mean/day (SD)	13.2 (10.8)	26.6 (21.8)	<0.001	24.22 (16.72)	26.1 (15.6)	0.004	8.3 (7.4)	9.1 (7.7)	0.218
2016; mean/day (SD)	11.6 (8.02)	25.9 (21.2)	<0.001	19.3 (10.0)	23.6 (14.5)	<0.001	7.6 (8.5)	8.2 (8.4)	0.272
2017; mean/day (SD)	11.4 (8.5)	25.6 (21.8)	<0.001	21.0 (13.2)	24.8 (16.7)	<0.001	7.5 (7.9)	8.4 (8.1)	0.108
Total; mean/day (SD)	12.6 (9.2)	28.4 (24.3)	<0.001	21.7 (14.0)	29.0 (19.4)	<0.001	7.4 (8.8)	7.9 (8.8)	0.009
1st quartile	6.6	12.2	<0.001	12.5	16.9	<0.001	1.2	1.6	0.009
Daily median	9.9	20.0	<0.001	18.1	24.0	<0.001	7.2	7.7	0.009
3rd quartile	15.5	37.4	<0.001	27.0	35.0	<0.001	14.6	15.3	0.009
IQR	9.0	25.2	<0.001	14.5	18.0	<0.001	13.4	13.7	0.009
Exceed daily mean WHO guideline; N (%)	110 (8.4)	908 (40.7)	<0.001	139 (4.2)	345 (9.8)	<0.001	N/A	N/A	N/A

IRQ, interquartile range; N/A, not applicable, N/D, no data; PM_2.5_, particulate matter with a diameter of 2.5 µm or less, PM_10_, particulate matter with a diameter of 10 µm or less; SD, standard deviation; Temp., temperature; WHO, World Health Organization.

**Table 4:** Spearman correlation between psychical variables (left part of the chart–Suwałki, right–Łomża).

**PM_2.5_** **µg/m^3^**	r = 0.518; *p* < 0.001	r = −0.608; *p* < 0.001
r = 0.668; *p* < 0.001	**PM_10_** **µg/m^3^**	r = −0.303; *p* < 0.001
r = −0.268; *p* < 0.001	r = −0.243; *p* < 0.001	**Temperature** **°C**

**Table 5:** Time-stratified case-crossover model for study population. The odds ratio of mortality with interquartile-range increase in exposure to air pollutants and temperature.

	Variables		Suwałki		Łomża		Ratio of Odds Ratio	
			OR (95% CI)	*p*	OR (95% CI)	*p*	ROR (95% CI)	*p*
Total mortality	LAG 0	PM_2.5_	1.044 (1.001–1.089)	0.04	1.061 (1.017–1.105)	0.006	0.984 (0.972–1.044)	0.29
		PM_10_	1.024 (0.995–1.054)	0.10	1.018 (0.991–1.047)	0.21	1.005 (0.966–1.047)	0.38
	LAG 1	PM_2.5_	1.027 (0.981–1.075)	0.27	1.029 (0.988–1.071)	0.17	0.998 (0.339–1.061)	0.48
		PM_10_	1.006 (0.978–1.036)	0.66	1.028 (1.000–1.058)	0.049	0.977 (0.939–1.017)	0.14
	LAG 2	PM_2.5_	1.005 (0.961–1.052)	0.83	1.036 (0.995–1.078)	0.82	0.971 (0.913–1.032)	0.17
		PM_10_	1.034 (1.005–1.064)	0.02	1.030 (1.001–1.060)	0.04	1.004 (0.965–1.045)	0.43
Cardiovascular mortality	LAG 0	PM_2.5_	1.085 (1.005–1.171)	0.04	1.086 (1.020–1.156)	0.01	0.999 (0.905–1.103)	0.50
		PM_10_	1.056 (1.006–1.107)	0.03	1.022 (0.979–1.067)	0.33	1.033 (0.972–1.098)	0.15
	LAG 1	PM_2.5_	1.034 (0.957–1.116)	0.39	1.029 (0.967–1.095)	0.37	1.005 (0.910–1.109)	0.46
		PM_10_	1.004 (0.957–1.054)	0.86	1.034 (0.991–1.080)	0.13	0.971 (0.909–1.036)	0.19
	LAG 2	PM_2.5_	1.014 (0.939–1.094)	0.73	0.992 (0.932–1.056)	0.80	0.981 (0.898–1.071)	0.33
		PM_10_	1.025 (0.977–1.076)	0.31	1.008 (0.965–1.053)	0.72	1.017 (0.952–1.085)	0.31
Pulmonary mortality	LAG 0	PM_2.5_	1.161 (0.987–1.365)	0.072	1.130 (0.967–1.320)	0.12	1.027 (0.821–1.286)	0.41
		PM_10_	1.023 (0.916–1.141)	0.68	1.011 (0.906–1.128)	0.87	1.012 (0.866–1.181)	0.44
	LAG 1	PM_2.5_	1.040 (0.885–1.221)	0.64	1.163 (1.021–1.380)	0.03	0.894 (0.717–1.115)	0.16
		PM_10_	0.979 (0.879–1.091)	0.69	1.013 (0.904–1.135)	0.82	0.966 (0.826–1.131)	0.33
	LAG 2	PM_2.5_	0.898 (0.759–1.062)	0.21	1.073 (0.921–1.251)	0.37	0.837 (0.667–1.050)	0.06
		PM_10_	0.951 (0.850–1.064)	0.38	1.044 (0.933–1.168)	0.45	0.911 (0.779–1.064)	0.12

PM_2.5_, particulate matter with a diameter of 2.5 µm or less; PM_10_, particulate matter with a diameter of 10 µm or less; Temp., temperature.
